# Important work demands for reducing sickness absence among workers with neck or upper back pain: a prospective cohort study

**DOI:** 10.1186/s12891-019-2909-1

**Published:** 2019-11-10

**Authors:** Stefan Oliv, Ewa Gustafsson, Adnan Noor Baloch, Mats Hagberg, Helena Sandén

**Affiliations:** Department of Occupational and Environmental Medicine, University of Gothenburg and Sahlgrenska University Hospital, Box 414 40530, Göteborg, Sweden

**Keywords:** Work ability, WAI, Occupation, Occupational health

## Abstract

**Background:**

The aim of this study was to investigate what exposure to work demands, physical and psychosocial, is associated with lower levels of sickness absence among workers with neck or upper back pain in different groups, by age, gender, duration of sickness absence and work ability score.

**Methods:**

This study was a prospective study of 4567 workers with neck or upper back pain. Data on neck or upper back pain, work demand and work ability were obtained from the Swedish Work Environment survey over a 3–year period (2009–2013). Register data on sickness absence, 1 year after each survey was conducted, were obtained from the Swedish health insurance database. Analyses were performed to estimate the association between self-reported work demands and registered sick days > 14 days. The analyses were stratified for gender, age group and work ability score.

**Results:**

Lower numbers of sick days were found for workers reporting low exposure to lifting ≥15 kg and twisted or forward-leaning work postures. Lower numbers of sick days were found for workers reporting high work control and seated work. The associations were generally stronger in the older age groups for the physical work demands.

**Conclusions:**

The findings in this study suggest that certain physical work demands and having high control over one’s work can result in lower sickness absence, especially among middle-aged and older workers with neck or upper back pain.

## Background

Neck pain is one of the most common musculoskeletal disorders, which causes sickness absence and early retirement [1–4]. Manual labour, awkward postures and repetitive work are commonly reported as causes for work-related neck disorders. Psychosocial factors such as high job demands, low support from supervisors and co-workers and low job control have also been reported as important contributors to musculoskeletal disorders [5–8]. Regardless of the cause, neck pain can lead to reduced work ability, reduced productivity, work disability and early retirement [9–11]. It has been shown that workers with high levels of physical work demands have a higher risk of work-related disability compared with workers in less physically demanding jobs [12, 13]. Women have a higher prevalence of neck pain compared with men, which is partly explained by differences in work exposure between men and women in that women more often perform repetitive work and report poor ergonomics to a higher degree then men [14–16]. In general, the peak prevalence of neck pain is at about age 45 years, which means that neck pain is most prevalent during working age [17]. Prevalence estimates differs across studies, a review found that the annual prevalence of neck pain in workers ranged from 27 to 48% [18]. Among workers, 11–14% report activity limitation due to neck pain as measured with compensation claims, but it has been suggested that this is a significant underestimation [19]. In Sweden approximately 23% of workers report neck pain during the last 3 months. Of those reporting neck pain 63% were women [20].

Recent studies have shown that workers with pain report lower work ability and also lower work performance and productivity [21, 22] compared with workers without pain. Self-reported work ability has also been shown to predict sickness absence in that workers who report a lower level of work ability are at higher risk for future sickness absence [23, 24].

In a previous study we investigated the association between physical and psychosocial work demands and work ability among workers with neck pain [25]. In that study it was found that excellent work ability was associated with having lower physical work demands among older workers. In the present study we wanted to examine the effect of work demands and work ability on future sickness absence.

The aim of this study was to investigate what exposure to work demands, physical and psychosocial, is associated with lower levels of sickness absence among workers with neck or upper back pain in different groups, by age, gender, length of sickness absence and work ability score.

## Methods

This was a follow-up study using data sourced from the Swedish Work Environment survey from 2009, 2011 and 2013, and from the Longitudinal integration database for health insurance and labour market studies (LISA) database from 2010, 2012 and 2014. All Swedish residents are included in the LISA database. Individuals in these databases were linked using the Swedish personal number in Statistics Sweden’s (SCB) Microdata Online Access (MONA) system. The Work Environment survey is an addition to the annual Swedish Labour Force survey.

### Source population

The Work Environment survey is conducted both by telephone interview and by postal questionnaire. During the telephone interview general questions about the work environment are asked, and also a question on work ability. A questionnaire is sent to those that agree to participate after the interview. The persons eligible for inclusion in the Work Environment survey are drawn from a representative sample of the Swedish population age 16–74 and inclusion criteria are employment and not on long-term sick or maternity leave. The Work Environment Questionnaire sent consisted of 121 questions regarding various aspects of work environment.

### Neck or upper back pain

Pain in the neck or upper back was measured using a self-reported question from the SCB Work Environment survey, asking about pain in the “upper back or neck” after work during the last 3 months. For this study, “pain in the neck or upper back” was defined as self-reported pain in the neck or upper back “a few days per week (1 day out of 2)” or more often [25].

### Work ability

In the interview part of the Work Environment survey a question is asked about work ability. The question which is asked is called the Work Ability Score (WAS) “Assume that your work ability at its best has a value of 10 points. How many points would you give your current work ability?” with a score of 1–10. Studies have shown that the WAS question has good validity and reliability when compared with the Work Ability Index (WAI) [24, 26]. In this study work ability as measured by the WAS was categorized in four categories, poor, moderate, good excellent work ability [27]. This question was included, as it were hypothesized that sickness absence would be different in the different WAS categories.

### Physical exposure

Exposure to physical work demands were measured using self-report questions from the Work Environment survey. The questions used for this study were questions regarding whole body and hand/arm vibrations, lifting > 15 kg, frequent rotations of the trunk, work in a twisted or bent position, work while leaning forwards without support, work with hand at or above shoulder level, repetitive movements and seated work. A detailed description of the questions can be found in a previous publication [25]. For this study, physical exposure was classified as *high exposure* for those that reported exposure “half of the time” or more and those who reported exposure less than half of the time were classified as having *low exposure* to that work demand. This exposure level has previously been used in similar studies [25, 28].

### Psychosocial exposure

Exposure to psychosocial work demands were made by indexing several self-report questions from the Work Environment survey. The variables used for psychosocial exposure in this study were the index for work demand, control and support. These variables were created by the SCB by indexing the answers for several questions into *high* or *low*. The index for demand was calculated based on four questions regarding: work stress, work attention, concentration and work load. The index for control was calculated using four questions regarding: work tempo, work organization, work planning and work influence. The support index was calculated using two questions regarding: support form supervisors and support from fellow workers. A detailed description of the questions and the index calculations can be found in a previous publication [25].

The measure for sickness absence used was obtained from the LISA database. The measure used was net sick days (n-days) > 14 days during the year following participation in the Work Environment survey. (Two days on 50% sickness absence were counted as one n-day.) A more detailed description can be found in a previous publication [27].

### Statistics

For all analyses in this study, SAS version 9.3 (SAS Institute, Cary, NC, USA) was used. Descriptive data on the neck or upper back pain and no neck or upper back pain groups were derived through frequency analyses. Wilcoxon Sum Rank Test was performed to estimate the association between high or low exposure to physical and psychosocial work demands and sickness absence as measured by n-days [29]. A difference between high and low work demands was considered significant when *P* < 0.05. The analyses were stratified for gender, age group and WAS. Quantile regression is a semi-parametric statistical method that investigates the difference between high and low work demands and several percentiles of sickness absence [30]. A quantile regression analysis was used to estimate the association between high or low exposure to different work demands and sickness absence (n-days) in the 90th, 95th and 99th percentiles. Coefficients from quantile regression are interpreted similarly to coefficients of ordinary linear regression, except that a quantile regression coefficient indicates the change in the value at the given percentile, not the mean, of the outcome variable.

## Results

### Study population

A total of 29,682 workers were sent the Work Environment Survey questionnaire and 18,786 replied (63% response rate). Four thousand five hundred sixty-seven workers reported neck or upper back pain after work “a few days per week (1 day out of 2)” or more often during the last 3 months. A larger proportion of women workers, 66%, compared to men workers reported neck or upper back pain (Table 1). The most common occupations among workers with neck or upper back pain were for women workers service, care and shop sales, and for men workers craft and related, trades, mining and construction (Table 2). Women workers had a higher mean number of n-days > 14 days compared to men workers (11 and 9 n-days, respectively). This pattern was also seen on the 90th and 95th percentiles. The highest number of n-days was found among workers who reported poor work ability (WAS 1–5). Higher numbers of sick days were found in older workers compared to younger (Table 3).

**Table 1:** Characteristics of the source population, 18,786 workers with or without neck or upper back pain (neck pain), (18,450 reported Work Ability Score). N = number of workers

	Women		Men	
	No neck pain	Neck pain	No neck pain	Neck pain
	*N* = 6957	*N* = 3023	*N* = 7262	*N* = 1544
Age category				
16–29 years	948	383	937	140
30–49 years	3285	1415	3534	711
50–64 years	2724	1225	2791	693
Work Ability Score				
WAS 10 Excellent	3960	1314	3822	574
WAS 8–9 Good	2139	1004	2529	566
WAS 6–7 Moderate	579	443	656	273
WAS 1–5 Poor	170	197	137	87

**Table 2:** Occupational groups of the source population, 18,786 workers with or without neck or upper back pain (neck pain). N = number of workers

Occupation	Women		Men	
	No neck pain	Neck pain	No neck pain	Neck pain
	% (*N* = 6957)	% *(N* = 3023)	% (*N* = 7262)	% (*N* = 1544)
Service, care and shop sales workers	25	28	7	8
Technicians and associated professionals, nurses	26	25	23	17
Professionals, e.g. teachers, computer technicians	25	21	21	17
Clerks, office, warehouse workers	11	12	4	4
Plant and machine operators	2	3	14	20
Craft and related, trade workers, miners, construction workers	1	1	17	24
Managers, legislators, senior officials	5	3	8	4
Elementary occupations, janitors, cleaners, etc.	4	5	2	3
Skilled agricultural, forestry and fishery workers	1	1	2	2
Other	< 1	1	2	2

**Table 3:** Sickness absence in one year: registered sickness-absence net days (n-days) > 14 days among the study population *N* = 4567 workers with self-reported neck or upper back pain by gender, age group and work ability score (WAS). Mean number of days, number of days on the 90th and 95th percentile (Pctl). *N* = number of workers

	Mean n-days (> 14 days)	90th Pctl n-days (> 14 days)	95th Pctl n-days (> 14 days)
Women	11	22	68
Men	9	11	50
16–29 years	4	1	22
30–49 years	10	17	63
50–64 years	13	24	84
WAS 10 Excellent	7	6	34
WAS 8–9 Good	10	15	51
WAS 6–7 Moderate	14	36	83
WAS 1–5 Poor	33	107	243

There was a difference in sick days found between the groups reporting low or high exposure to several physical work demands. The groups reporting low exposure to: lifting ≥15 kg, twisted work posture, leaning forward without support and frequent trunk rotations had fewer n-days. Among men, a difference was found for low exposure compared to high exposure, to whole-body vibrations and working with hands above shoulder level (Tables 4 and 5). For women, reporting high control was associated with fewer sickness absence days compared to having low control (Table 6). Those who reported high exposure to seated work had fewer registered sickness absence n-days (> 14 days) compared to the group reporting low exposure to seated work. Also, the group who reported high control over one’s work had fewer registered sickness absence n-days (> 14 days) compared to the group reporting low control (Tables 4, 5 and 6). The analysis divided on age groups found differences mainly in the middle and older age groups, except for having high control, where differences were found for both the youngest and oldest age groups (Tables 7 and 8).

**Table 4:** One year registered sickness-absence net days (n-days) > 14 days for groups reporting high or low physical work demand exposure

	Work demand exposure				
	Low		High		
	*N*	Mean n-days	*N*	Mean n-days	P*
Lifting ≥15 kg					
All	3054	9	1487	13	**< 0.01**
Women	2226	10	783	15	**< 0.01**
Men	828	8	704	11	**< 0.05**
16–29 years	311	3	210	6	0.62
30–49 years	1443	9	677	13	**< 0.01**
50–64 years	1300	12	600	16	**0.01**
Frequent trunk rotations					
All	2325	8	2203	13	**< 0.01**
Women	1583	9	1421	14	**< 0.01**
Men	742	8	782	11	**< 0.05**
16–29 years	195	4	325	5	0.74
30–49 years	1188	7	928	14	**< 0.01**
50–64 years	942	11	950	15	**< 0.05**
Twisted work posture					
All	3350	10	1151	13	**< 0.01**
Women	2271	11	711	13	**0.01**
Men	1079	8	440	13	**< 0.01**
16–29 years	321	3	195	6	0.31
30–49 years	1608	9	494	13	**< 0.01**
50–64 years	1421	12	462	15	0.05
Hand held vibrating tools					
All	4214	11	308	12	0.90
Women	2919	11	78	16	0.81
Men	1295	9	230	11	0.37
16–29 years	486	3	33	17	0.32
30–49 years	1957	10	153	11	0.89
50–64 years	1771	13	122	12	0.45
Leaning forward without support					
All	3312	9	1201	14	**< 0.01**
Women	2245	10	747	14	**< 0.01**
Men	1067	8	454	14	**< 0.01**
16–29 years	326	3	190	7	0.68
30–49 years	1591	9	521	15	**< 0.01**
50–64 years	1395	12	490	16	0.07

Four thousand five hundred sixty-seven workers with self-reported neck or upper back pain by gender and age group. Mean number of n-days for description only. *P* values indicate the difference in *distribution* of absence days between reporting high or low exposure. Wilcoxon Rank Sum Test * = two tailed; bold numbers indicate a statistically significant difference. N = number of workers

**Table 5:** One year registered sickness-absence net days (n-days) > 14 days for groups reporting high or low physical work demand exposure

	Work demand exposure				
	Low		High		
	*N*	Mean n-days	*N*	Mean n-days	P*
Hands at shoulder level or higher					
All	3782	10	736	11	0.18
Women	2597	11	395	12	0.56
Men	1185	9	341	10	**< 0.05**
16–29 years	398	4	121	6	0.38
30–49 years	1788	10	321	12	**< 0.05**
50–64 years	1596	13	294	12	0.68
Whole-body vibrations					
All	4245	10	283	17	0.27
Women	2959	11	48	23	0.64
Men	1286	8	235	15	**< 0.05**
16–29 years	486	4	34	12	0.77
30–49 years	1978	10	134	18	0.07
50–64 years	1781	13	115	16	0.94
Repetitive movements					
All	2873	10	1625	11	0.68
Women	1956	11	1021	12	0.62
Men	917	9	604	10	0.06
16–29 years	273	3	246	5	0.98
30–49 years	1405	9	693	11	0.61
50–64 years	1195	13	686	13	0.65
Seated work					
All	1970	13	2558	9	**< 0.01**
Women	1287	14	1712	10	**< 0.01**
Men	683	11	846	9	**< 0.01**
16–29 years	262	5	256	3	0.21
30–49 years	855	12	1260	9	**< 0.01**
50–64 years	853	15	1042	11	**< 0.05**

Four thousand five hundred sixty-seven workers with self-reported neck or upper back pain by gender and age group. Mean number of n-days for description only. *P* values indicate the difference in *distribution* of sick days between reporting high or low exposure. Wilcoxon Rank Sum Test * = two tailed; bold numbers indicate a statistically significant difference. N = number of workers

**Table 6:** Difference in distribution of one year registered sickness-absence net days (n-days) > 14 days by reporting high or low psychosocial work demand exposure

	Work demand exposure				
	Low		High		
	*N*	Mean n-days	*N*	Mean n-days	P*
Demand					
All	1619	9	2930	11	0.35
Women	983	9	2032	13	0.07
Men	636	10	898	9	0.10
16–29 years	189	2	333	5	0.30
30–49 years	758	9	1364	11	0.24
50–64 years	672	11	1233	14	0.80
Support					
All	2097	11	2452	11	0.29
Women	1294	12	1721	11	0.17
Men	803	9	731	10	0.64
16–29 years	212	6	310	3	0.42
30–49 years	921	10	1201	10	0.45
50–64 years	964	12	941	14	0.95
Control					
All	2473	13	2076	8	**< 0.01**
Women	1787	14	1228	7	**< 0.01**
Men	686	8	848	10	0.70
16–29 years	320	6	202	2	**< 0.05**
30–49 years	1103	12	1019	8	0.06
50–64 years	1050	15	855	10	**< 0.01**

Four thousand five hundred sixty-seven workers with self-reported neck or upper back pain by gender and age group. Mean number of n-days for description only. *P* values indicate the difference in *distribution* of sick days between reporting high or low exposure. Wilcoxon Rank Sum Test * = two tailed; bold numbers indicate a statistically significant difference. *N* = number of workers

**Table 7:** Difference in distribution of one year registered sickness-absence net days (n-days) > 14 days by reporting high or low physical work demand exposure

	Work demand exposure				
	Low		High		
	*N*	Mean n-days	*N*	Mean n-days	P*
Lifting ≥15 kg					
WAS 10 Excellent	1344	6	534	10	**< 0.01**
WAS 8–9 Good	1038	8	522	12	0.06
WAS 6–7 Moderate	415	14	298	14	0.10
WAS 1–5 Poor	179	36	103	29	0.83
Twisted work posture					
WAS 10 Excellent	1423	6	446	9	0.41
WAS 8–9 Good	1173	9	372	13	**< 0.01**
WAS 6–7 Moderate	469	11	234	17	**< 0.05**
WAS 1–5 Poor	206	37	72	22	0.43
Handheld vibrating tools					
WAS 10 Excellent	1763	7	111	8	0.50
WAS 8–9 Good	1442	9	111	12	0.56
WAS 6–7 Moderate	650	14	59	11	0.19
WAS 1–5 Poor	261	32	19	45	0.63
Leaning forward without support					
WAS 10 Excellent	1426	6	444	9	**< 0.01**
WAS 8–9 Good	1146	8	403	14	**< 0.01**
WAS 6–7 Moderate	474	13	232	15	0.16
WAS 1–5 Poor	193	33	88	32	0.87
Hands at shoulder level or higher					
WAS 10 Excellent	1607	7	265	7	0.40
WAS 8–9 Good	1315	10	237	10	0.18
WAS 6–7 Moderate	562	16	143	10	0.19
WAS 1–5 Poor	218	33	63	33	0.84
Whole-body vibration					
WAS 10 Excellent	1776	7	99	11	0.29
WAS 8–9 Good	1451	9	105	17	0.43
WAS 6–7 Moderate	656	14	55	11	0.93
WAS 1–5 Poor	267	30	13	94	0.31
Frequent trunk rotations					
WAS 10 Excellent	1057	5	817	9	**< 0.05**
WAS 8–9 Good	797	8	760	11	0.08
WAS 6–7 Moderate	312	10	397	16	0.07
WAS 1–5 Poor	111	33	170	33	0.90
Repetitive movements					
WAS 10 Excellent	1241	7	621	6	0.42
WAS 8–9 Good	988	8	559	10	0.41
WAS 6–7 Moderate	421	13	282	14	0.53
WAS 1–5 Poor	162	31	117	36	0.20
Seated work					
WAS 10 Excellent	712	9	1159	5	**< 0.01**
WAS 8–9 Good	688	12	872	7	**< 0.01**
WAS 6–7 Moderate	367	13	343	14	0.82
WAS 1–5 Poor	154	26	125	42	0.18

Four thousand five hundred sixty-seven workers with self-reported neck or upper back pain by Work Ability Score (WAS). Mean number of n-days for description only. *P* values indicate the difference in *distribution* of sick days between reporting high or low exposure. Wilcoxon Rank Sum Test * = two tailed; bold numbers indicate a statistically significant difference. N = number of workers

**Table 8:** Difference in one year registered sickness-absence net days (n-days) > 14 days by reporting high or low psychosocial work demand exposure

	Work demand exposure				
	Low		High		
	*N*	Mean N-days	*N*	Mean n-days	P*
Support					
WAS 10 Excellent	798	5	1084	8	0.84
WAS 8–9 Good	716	9	847	9	0.69
WAS 6–7 Moderate	389	15	324	13	0.72
WAS 1–5 Poor	149	34	133	32	0.85
Demand					
WAS 10 Excellent	638	6	1244	7	0.37
WAS 8–9 Good	563	9	1000	10	0.87
WAS 6–7 Moderate	267	10	446	16	0.23
WAS 1–5 Poor	114	28	168	36	0.25
Control					
WAS 10 Excellent	982	8	900	6	0.35
WAS 8–9 Good	862	11	701	8	**< 0.05**
WAS 6–7 Moderate	418	16	295	11	0.05
WAS 1–5 Poor	148	42	134	22	**< 0.05**

Four thousand five hundred sixty-seven workers with self-reported neck or upper back pain by Work Ability Score (WAS). *P* values indicate the difference in *distribution* of sick days between reporting high or low exposure. Wilcoxon Rank Sum Test * = two tailed; bold numbers indicate a statistically significant difference. N = number of workers

The quantile regression analysis showed a difference in lower number of n-days on the 90th percentile for those workers who reported low exposure to lifting ≥15 kg (14 compared to 28 n-days), twisted work posture (15 compared 26 n-days), leaning forward without support (14 compared to 34 n-days) and frequent trunk rotations (12 compared to 24 n-days). There was a lower number of n-days on the 90th percentile for those workers reporting high exposure to seated work (13 compared to 27 n-days) and for high control (13 compared to 24 n-days).

On the 95th percentile there was a lower number of sickness absence days among workers reporting low exposure to lifting ≥15 kg (50 compared to 89 n-days), twisted work posture (54 compared 73 n-days), whole-body vibrations (71 compared to 130 n-days), leaning forward without support (51 compared to 94 n-days) and frequent trunk rotations (42 compared to 84 n-days). There was a lower number of n-days on the 90th percentile for those workers reporting high exposure to seated work (45 compared to 86 n-days) and for high control (40 compared to 83 n-days).

On the 90th percentile there was a lower number of sickness absence days among workers reporting low exposure to frequent trunk rotations (199 compared to 297 n-days).

## Discussion

The main findings in this study suggest that low or high exposure to certain work demands, such as low exposure to lifting > 15 kg, twisted/bent work postures, high exposure to seated work and high control, can result in lower sickness absence for workers with neck or upper back pain. A difference, by reporting high or low exposure, in sickness absence was found among those who reported low exposure to several physical work demands and those who reported high control of their work and high exposure to seated work. These differences were mainly found in the middle and older age groups.

In this study we used the WAS as a health measure. The group who reported excellent work ability (WAS 10) and also reported low exposure to physical work demands (leaning forward without support and frequent twisting) had n-days compared to those who reported high exposure. The excellent work ability group also had fewer sickness absence days if reporting high exposure to seated work. The only finding in the group with poor work ability (WAS 1–5) was that those who reported high control over their work had fewer sickness absence days than those reporting low control. These findings are somewhat supported by a Finnish study [31], where it was also found that workers reporting lower levels of work ability have higher numbers of sickness absence days regardless of age, sex or occupation. In this study, the measure of neck or upper back pain consisted of a question regarding whether the worker had had pain in the “upper back or neck” after work during the last 3 months, 2 days per week or more often. A study by Holtermann et al. [4] used a 0–9 scale to describe pain intensity, with 0 being no pain and 9 being the worst pain possible. In that study it was found that among workers with a pain intensity score ≥ 7, 23% had long-term sickness episodes compared with 15% among those who reported a score of 4 on pain intensity.

Reporting high or low exposure to the work demands measured in this study gave different results in the different age groups. In the youngest age group (16–24 years) we found a difference between reporting high control over one’s work and a lower number of sickness absence days compared to those who reported low control. In the oldest age group (50–54 years) there were also a lower number of n-days among those reporting high control but also reporting high exposure to seated work and low exposure to frequent twisting and lifting ≥15 kg. Previous studies [31, 32] investigating sickness absence in different age groups also found the highest numbers of sickness absence among older workers, and furthermore that both musculoskeletal impairment and self-reported work ability and stressful work were determinants of future sickness absence. In this study we also found that low exposure to physical work demands is associated with lower numbers of sickness absence days in the middle-aged group.

The measured exposure to work demands showed a similar pattern for both women and men workers with a few exceptions. Among women, there was a difference in sickness absence days between reporting high or low control over one’s work. Among men there were lower numbers of n-days among those reporting low exposure to whole-body vibration and working with hands at shoulder level or higher, but not among women. However, there were few women who reported high exposure to these work demands, which could affect the results. As found in previous studies, women workers had generally higher numbers of sickness absence days than men. This is in line with previous studies which have found higher risk of disability pensions among women workers [2].

The quantile regression analysis was used to investigate the effect of work demands on different lengths of sickness absence. The analysis showed that high or low exposure to the different work demands affect the level of sickness absence mainly on the 90th and 95th percentile but not on the 99th (except for exposure to frequent rotations). This can be interpreted to mean that exposure to work demands is linked to sickness absence for shorter but not long-term sickness absence. A previous study divided sickness absence into three time periods, early (≤14 days), medium–late (15–90 days) and late (≥90 days) for return to work, using the same data source on registered sickness absence as in this study [33]. If applied to this study, exposure to several work demands affects sickness absence for short (early) and medium long (medium–late) periods, but only one work demand (frequent rotations) affects longer (late) periods of sickness absence. This analysis also shows the difference in numbers of sickness absence days; for instance, the group that reported low exposure to heavy lifting had 39 fewer days compared to the high exposure on the 95th percentile. The group having high control over their work had 43 fewer days of sickness absence than the group with low control.

### Strengths, limitations and methodological considerations

This study has several strengths. It is prospective, it is based on a representative sample of the Swedish working population and it includes sickness absence from official registries. Some weaknesses of the study include the use of self-reporting by means of telephone interview and questionnaire. As there is no objective measure (except sickness absence), we cannot appraise the seriousness of the disorders or the exact level of exposure to the different work demands. No measurements of the intensity of the neck or upper back pain were made. This is a limitation in this study, as it is known that intensity of the neck pain is a predictor for long-term sick leave [4]. There are some evidence that multisite pain can have a larger impact on sickness absence than single site pain [34]. In this study we have only included workers who reported neck or upper back pain and we do not know if they also have pain in other body sites. We also had no information about other confounders, including socio-demographic or individual factors such as self-efficacy, which also are known factors that influence sickness absence [35]. The registry measure of sickness absence from the LISA registry covers all causes of sickness absence, and in this study, we cannot distinguish between different causes. A methodological aspect of this study is that it took place in Sweden. Very few studies of sickness absence have investigated whether the national context plays a role in the results [36].

## Conclusions

The findings in this study suggest that certain physical work demands and having high control over one’s work can result in lower sickness absence, especially among middle-aged and older workers with neck or upper back pain.

## Data Availability

The data used for this study is available through Statistics Sweden.

## Abbreviations

LISA: Longitudinal integration database for health insurance and labour market studies
MONA: Microdata online access
N-days: Net days
SCB: Statistics Sweden
WAI: Work ability index
WAS: Work ability score
